# Phylogeographic Structure of the White-Footed Mouse and the Deer Mouse, Two Lyme Disease Reservoir Hosts in Québec

**DOI:** 10.1371/journal.pone.0144112

**Published:** 2015-12-03

**Authors:** Jessica Fiset, Nathalie Tessier, Virginie Millien, Francois-Joseph Lapointe

**Affiliations:** 1 Département de sciences biologiques, Université de Montréal, Montréal, Québec, Canada; 2 Ministère des Forêts, de la Faune et des Parcs, Longueuil, Québec, Canada; 3 Redpath Museum, McGill University, Montréal, Québec, Canada; University of Missouri, UNITED STATES

## Abstract

Modification of a species range is one of many consequences of climate change and is driving the emergence of Lyme disease in eastern Canada. The primary reservoir host of the bacteria responsible for Lyme disease, *Borrelia burgdorferi*, is the white-footed mouse (*Peromyscus leucopus)*, whose range is rapidly shifting north into southern Québec. The deer mouse, *P*. *maniculatus*, is occurring over most Québec province and is a less competent host for *B*. *burgdorferi*. Here, we compared the phylogeographic structure of both *Peromyscus* species in Québec. Using a combination of multiple mitochondrial DNA markers and phylogeographic methods, we detected an ongoing and rapid expansion of *P*. *leucopus*, while *P*. *maniculatus* appears more stable. Haplotype and populations networks indicated that populations of *P*. *maniculatus* exhibit more genetic structure than *P*. *leucopus* across the study area. Furthermore, significant and consistent genetic divergences between populations of the two species on both sides of the St. Lawrence River suggest that distinct lineages of *P*. *leucopus* and *P*. *maniculatus* with different ancestral origins colonized Southern Québec following the Last Glacial Maximum. The phylogeographic structure of pathogens is expected to mirror the structure observed in their reservoir hosts. As different strains of *Borrelia burgdorferi* may be associated with different levels of pathogenicity and immune responses of their hosts, our results are helpful at better understanding the pattern of spread of Lyme disease in a zone of emergence, and associated risk for human populations.

## Introduction

Climate change is occurring at a rate unprecedented in Earth history [1], and in the Northern Hemisphere, we are witnessing increasing global average temperature, shorter winters, and more abundant precipitations [2]. Species may alter their geographical range to track changes in environmental conditions. Most notably, climate change is thought to be the driver of northern range expansion or range shifts in a number of species, which in turn is affecting the composition of local terrestrial and aquatic communities [3–6]. A number of North American terrestrial species reach the northern limit of their geographic distribution around the Great Lakes and the St. Lawrence River [7]. Southern Québec thus represents an important transition zone and an ideal location for studying the pattern of range shift in response to global change [8,9].

Molecular markers have proven useful to reconstruct the pattern of range shift in response to global warming since the Last Glacial Maximum, but also to the most recent historical global change [10]. Phylogeographic studies allow to trace past geographical patterns of genetic differentiation among populations, and have proven particularly useful to identify historical vicariance events that have shaped current species distributions [10–11]. Here, we used such an approach and compared two closely related species of small mammal found across most of the Western United States and up into Southern Québec in Canada.

The deer mouse (*Peromyscus maniculatus*) and the white-footed mouse (*P*. *leucopus*) are closely related rodent species of the family Cricetidae [12–13] that have diverged approximately 500,000 years ago [14]. The two species are similar in their ecology and morphology, and occur in sympatry across most of the range of the white-footed mouse. *Peromyscus leucopus* reaches its northern distribution limit in Southern Québec [8, 15]. The range of *P*. *leucopus* has expanded northwards over the last few decades in the Michigan upper and lower Peninsula [9], as well as in Southern Québec [16]. The rate of poleward expansion was estimated at 10 to 15 km/year and in both regions, such a rapid rate of expansion was attributed to global warming [9,16]. *Peromyscus maniculatus* is a widespread species, ranging from Mexico to Northern Canada. It is a dominant component of small mammal communities and has been established in southern Québec for a much longer time than *P*. *leucopus* [17].

Previous studies have shown similar patterns in the phylogeography of *P*. maniculatus and *P*. *leucopus* in the United States. Namely, the pattern of genetic differentiation of the mtDNA control region (D-Loop) revealed three clades of *P*. *leucopus* that originated from distinct glacial refugia in North America. One of these clades corresponds to the east coast of the United States, while another clade is associated with the upper midwest [18, 19]. Six clades were identified for *P*. *maniculatus* across North America using Cytochrome B sequences [20]. There again, one clade was restricted to the east coast of the United States, while a second one corresponded to the upper Midwest plus Manitoba, Ontario and Québec in Canada.

Here, we propose to apply phylogeographic methods not only to assess past vicariance events, but rather to detect a genetic signature of a recent range expansion in *P*. *leucopus*. To do so, we used a combination of multiple mtDNA markers to resolve the phylogeography of *P*. *leucopus* and *P*. *maniculatus* in Southern Québec.

The use of mitochondrial DNA (mtDNA) has long been advocated in phylogeography for its rapid rate of evolution [10]. However, the use of several markers is strongly recommended, as a specific locus may be subjected to different evolutionary constraints, due to selection on that locus or simply by stochasticity [21]. The mtDNA markers most frequently used in phylogeographic studies of small mammals are the Cytochrome B (Cyt B), Cytochrome Oxidase III (COIII) and the control region (D-Loop) [19–20, 22–24]. Here, we combined these three highly polymorphic mtDNA markers with two others (ATP synthase 8 and 16S ribosomal RNA) in order to achieve better phylogeographic resolution. We applied a range of methods to evaluate whether expanding populations of white-footed mouse display a different phylogeographic structure compared to that of the deer mouse, a well-established species in Québec. More specifically, for both species we tested whether the phylogeographic patterns for different mtDNA markers were congruent within Québec and if major geographic barriers were limiting the dispersal of *P*. *leucopus*, as it has been previously shown [25, 26]. We also compared our results to previously published work to identify the North American clades associated with the populations from Québec and identify potential glacial refugia in both species.

The white-footed mouse is known to be the main reservoir host for the tick vector of *Borrelia burgdorferri*, a spirochete bacterium responsible for Lyme disease in North America [27, 28]. Lyme disease is an emerging zoonotic disease in North American and the risk for humans is rapidly increasing and expanding northwards into Southern Québec [29]. The white-footed mouse is an excellent reservoir for Lyme disease. Unlike other mammals such as dog, deer and human whose immune system fights the pathogen, *P*. *leucopus* remains an asymptomatic carrier throughout its life once infected [28]. The white-footed mouse is also able to efficiently transmit (90%) all strains of *Borrelia* to ticks [28, 30]. Yet, the deer mouse (*P*. *maniculatus*) is also competent to maintain and transmit *Borrelia* in areas where the white-footed mouse is not occurring [31].

Here, we conducted a comparative analysis of *P*. *leucopus*, a rapidly expanding and highly competent host, and *P*. *maniculatus*, a more established and less competent host. Our results will allow to better anticipate the geographic patterns of range expansion in the white-footed mouse into Southern Québec and adapt to the associated rapid spread of Lyme disease in this region located at the most northern limit of the distribution of *P*. *leucopus*.

## Materials and Methods

### Field sampling

**Fig 1:**
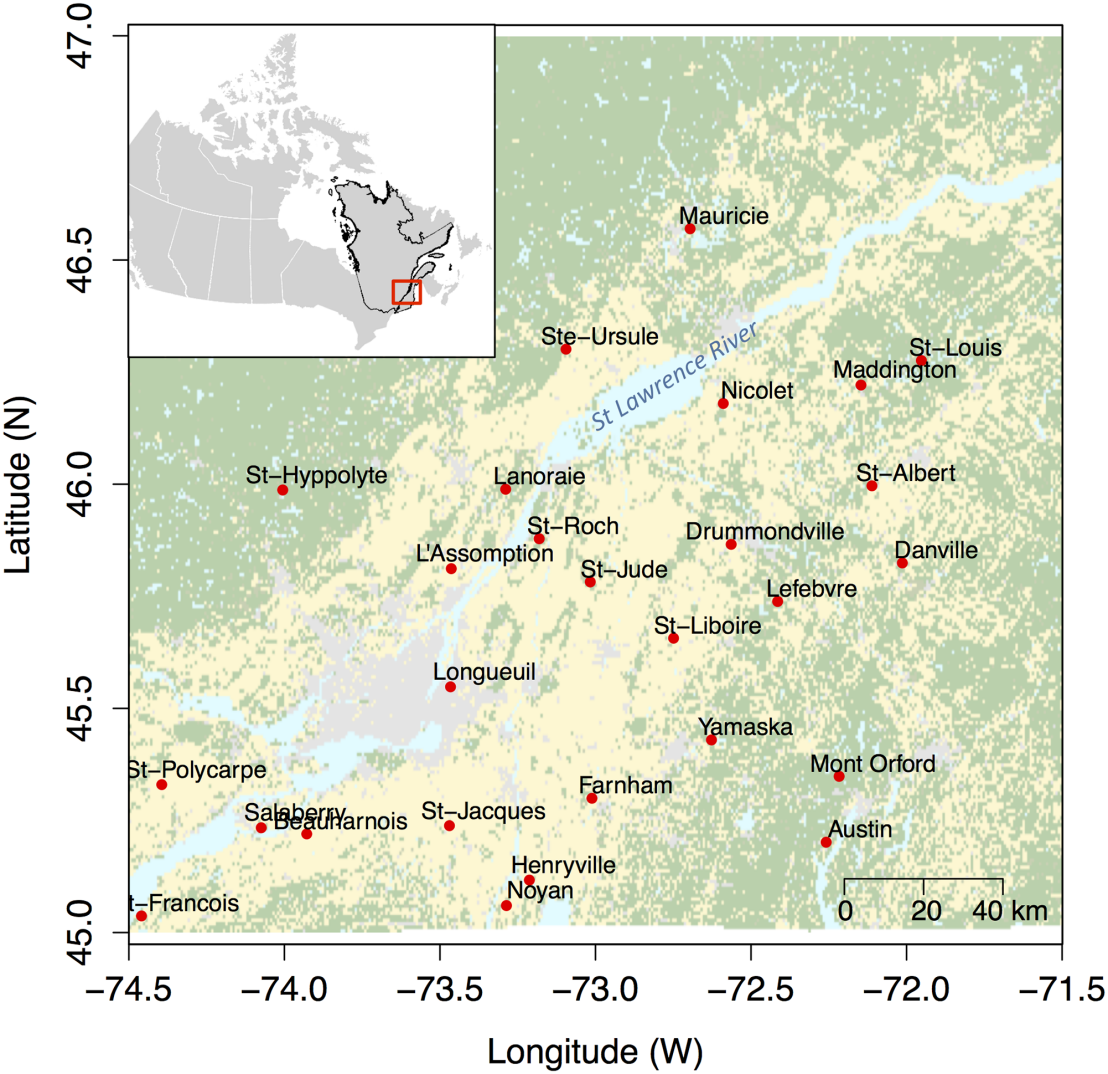
Location of the 26 study sites sampled during the summer 2011 in southern Québec. An additional site (Saguenay) is located further north-east and is not shown on this map.

The sampling took place at a total of 28 sites (Fig 1). A majority of these sites were located within the current known distribution of Lyme disease occurrence in Southern Québec, [27, 29, 32, 33]. The other sites were located in areas were *Borrelia* has not been detected but within the geographical range of *P*. *leucopus* in Québec (Fig 1). A total of 96 individuals of white-footed mice and 53 individuals of deer mice were captured in forest patches during the summer of 2011 using Sherman live traps (Table 1), of which respectively 87 and 43 specimens were successfully sequenced for all five mtDNA markers. All specimens were brought back to the laboratory for tissue sampling and genetic analyses.

**Table 1:** Geographical coordinates of the sampling sites, and sample size for *P*. *leucopus* (*P*. *l*.) and *P*. *maniculatus* (*P*. *m*.).

Site	Longitude (N)	Latitude (W)	*P*.*l*.	*P*.*m*.
Austin	-72.25	45.20	0	7
Danville	-72.02	45.81	0	1
Maddington	-72.14	46.21	0	3
Nicolet	-72.58	46.17	0	4
Mont-Orford	-72.21	45.34	0	8
St-Louis	-71.95	46.27	0	3
St-Albert	-72.10	45.99	0	2
Lanoraie	-73.29	45.99	0	9
Yamaska	-72.62	45.42	0	7
St-Hyppolyte	-74.00	45.98	0	1
Mauricie	-72.70	46.57	0	1
Saguenay	-71.06	48.42	0	5
Farnham	-73.01	45.30	4	1
Henryville	-73.21	45.11	6	1
Noyan	-73.28	45.05	7	0
Drummondville	-72.55	45.86	5	0
Lefebvre	-72.41	45.65	2	0
Longueuil	-73.46	45.54	10	0
St-François	-74.45	45.03	7	0
Beauharnois	-73.93	45.21	6	0
St-Jacques	-73.47	45.24	2	0
Sallaberry	-74.07	45.23	2	0
L’Assomption	-73.46	45.81	5	0
St-Jude	-73.01	45.77	7	0
St-Liboire	-72.74	45.65	14	0
St-Polycarpe	-74.39	45.32	10	0
St-Roch	-73.17	45.87	4	0
St-Ursule	-73.09	46.29	5	0

### Ethics Statement

All procedures were approved by the Québec Government (SEG permit #2011-05-15-014-00-S-F), the McGill University Animal Care Committee (AUP#5420) and the Comité de déontologie de l’expérimentation sur les animaux de l’Université de Montréal (#11–054).

### Species identification and sequencing

For each specimen, we extracted DNA from tail tissues using the standard Quick-Lysis protocol [34], and all specimens were identified to the species using species-specific primers in multiplex polymerase chain reaction (PCR) [25, 35]. We then amplified five mtDNA regions using the primers described in Table 2. The amplifications were carried out in a 50 μl volume comprising 5 μl of 10x reaction buffer, 4 μl of dNTP solution at 2.5 mM, 20pM of each primers, 1.5 u of Taq DNA polymerase and 50 to 300 ng of DNA. The DNA amplification protocol was 94°C for 1 min, 35 x (94°C for 45 sec, 54°C for 1 min 30 sec, 72°C for 45 sec) and 72°C for 7 min. The PCR products were then sequenced at the McGill University Genome Québec Innovation Centre. We visualised the chromatograms with the program 4Peaks 1.7 [36] and aligned the sequences with the program ClustalX 2.1 [37]. We removed ambiguous sections at the beginning and the end of each sequence, as well as the gaps and repetitions susceptible to be polymerase reaction errors. Sequence alignments for all markers combined are available as supplementary material for *P*. *leucopus* (S1 Table) and *P*. *maniculatus* (S2 Table).

**Table 2:** Primers used for amplifying the five mtDNA regions used in this study.

Region	Name	Strand	Sequences (5’-3’)	Reference
Control region (D-Loop)	L15926	Foward	CAATTCCCCGGTCTTGTAAACC	Kocher *et al*. 1989
	H16340	Reverse	CCTGAAGTAGGAACCAGATG	
	DL-PeroF	Foward	GTCTAATCAGCCCATGACCA	Designed in this study
	DL-PeroR	Reverse	AGCATTTTCAGTGCTTTGCT	
Cytochrome B	L14841	Foward	CCATCCAACATCTCCGCATGATGAAA	Kocher *et al*. 1989
	H15149	Reverse	CCCTCAGAATGATATTTGGCCTCA	
	L14553	Foward	CTACCATGAGGACAAATATC	Sullivan *et al*. 1997
	MOUSE-TR	Reverse	TTCCATTTYTGGTTTACAAGACCA	Wade 1999
ATP8	AP8-1F	Foward	GCATTAACCTTTTAAGTTAAAG	Johnson *et al*. 1998
	AP8-2R	Reverse	GGCGAATAGATTTTCGTTCA	
16S	16S-1F	Foward	GTGCAAAGGTAGCATAATCA	Johnson *et al*. 1998
	16S-4R	Reverse	TGTCCTGATCCAACATGAG	
COIII	L8618	Foward	TACGTATTCACCCTTCTAGTAAGCCT	Riddle 1995
	H9323	Reverse	ACTACGTCTACGAAATGTCAGTATCA	

### Data analysis

We first estimated numbers of haplotypes, private haplotypes, parsimony-informative sites, and segregating sites, as well as nucleotide diversity and haplotype diversity with DnaSP version 5.10.1 [38] and the R package ‘PopGenome’ version 2.1.0 [39]. For phylogeographic analysis, the optimal substitution model was then determined with the program ModelTest [40]. We reconstructed phylogeographic trees for each species and all five mtDNA markers separately with the R package ‘ape’ version 3.1–4 [41] using the neighbor-joining (NJ) algorithm [42] applied to pairwise genetic distances among individuals (function *nj*) and corrected with the K2P model [43] for unequal base frequencies [44]. In addition, PhyML [45] was also applied to the five mtDNA markers, based on the same substitution model [43], and using the NJ tree as a starting tree. For better visualization, the trees were artificially rooted on the longest branch (midpoint rooting [46]) with the R package ‘phangorn’ version 1.1–1 [47], and a majority-rule consensus was computed (function *consensus*) to identify the supported clades. The robustness of separate phylogeographical trees was assessed with bootstrap values [48] computed from 100 replicates, and tree resolution was measured by counting the ratio of non-zero length branches over the total number of branches. We performed an incongruence length difference (ILD) test [49] with PAUP* 4.0 [50], and test of congruence among distance matrix (CADM) [51, 52] with the R package ‘ape’ to compare the markers with one another and determine whether they led to congruent phylogeographic patterns (function *CADM*.*global*). We then ran a posteriori comparisons using Mantel tests [53] to test for the significance of differences between pairs of markers (function *CADM*.*post*).

We built haplotype networks with the program Haplotype Viewer [54]. Whereas a phylogeographic tree links individuals, a haplotype network links genetic sequences, thus merging all unique haplotypes at a single node. For both species, we performed a clanistic analysis to evaluate the clans and slices purity [55], where clans are groups obtained by cutting a single branch (bipartition), and slices are groups obtained by cutting two branches (tripartition) in an unrooted network. We calculated the clan and slice purity by counting the number of haplotypes from each sampling site in a group divided by the size of this group (a value of one indicating that a given clan—or slice—contained only haplotypes from a single locality). We also calculated the clan and slice diameter as the maximum distance (number of mutations) between two haplotypes from the same locality in a group; this value was standardized by the total diameter of the network for comparison purposes. We used a Student t-test on all clanistic variables to test for significant differences in the haplotype networks of the two *Peromyscus* species.

To further detect differences in the geographical pattern of genetic variability between the two species, we computed population networks among localities (nodes in the graph) by considering only shared haplotypes [56]. Namely, two populations (sites) are connected by an edge in the network only when they have at least one haplotype in common. To account for unequal sampling sizes, we estimated the connection probability with a resampling approach in which a single haplotype per site was randomly selected at each iteration prior to constructing the network. This procedure was repeated 1000 times to derive pairwise connection probabilities among sites, for each species and each marker. We used two indices to compare these population networks: the graph density (the number of connections in the graph relative to the number of possible connections), and the node degree distributions (where the degree of a node is the number of edges connected to that node). These two indices were compared between the two *Peromyscus* species with Student t-tests and non-parametric Mann Whitney tests, respectively.

We determined possible demographic changes in population size or selective regimes for both species independently as well as for both shores of the St. Lawrence River with three different neutrality test and computed mismatch distributions [57] to detect population expansion. To test for deviations from neutrality, we calculated Tajima’s D [58], Fu’s F_S_ [59], and Ramos-Onsins and Rozas’ R_2_ [60] statistics separately for the five markers, as well as for all markers combined. We assessed statistical significance of each test with 1000 replicates using either Arlequin version 3.5.2.1[61], DnaSP version 5.10.1 [38], or the R package ‘Pegas’ version 0.7 [62]. The mismatch distributions were computed using the function MMD with the R package ‘Pegas’ [62]. We also computed Fst-values among all pairs of populations and between shores for each species with all markers combined, and assessed statistical significance with 1000 permutations in Arlequin version 3.5.2.1 [61].

Finally, we estimated divergence times for the split separating the north and south shores of the St. Lawrence River for the two species using a Bayesian analysis and a relaxed-clock dating implemented in BEAST version 1.8.2 [63]. To do so, the 87 D-Loop sequences of *Peromyscus leucopus* included in the present study were combined with all sequences listed in Shipp-Pennock *et al*. [19], and the 43 Cyt B sequences of *Peromyscus maniculatus* were combined with those listed in Dragoo *et al*. [20] to determine phylogeographic clades corresponding to past vicariance events. In each case, the combined dataset was trimmed to compare sequences of equal lengths. To calibrate the molecular clock for each mtDNA marker, the corresponding sequences of *Peromyscus leucopus* (respectively *Peromyscus maniculatus*) were used as outgroup, and the split between both species was estimated at 3.5 Mya [64]. The analyses were run for 10,000,000 generations, sampling model parameters every 1000 generations and consensus trees were produced with TreeAnnotator v. 1.8.2 after elimination of 20% of the trees as burn-in. The resulting trees for both species were visualized in FigTree 1.4.2.

## Results

All markers exhibited similar levels of polymorphism and nucleotide diversity, except for the 16S region, which was the least variable (Table 3). All markers displayed different numbers of haplotypes between the two species, except for the ATP8, the only marker with a similar level of variability in both species. For all other four markers, *P*. *maniculatus* exhibited more haplotype diversity than *P*. *leucopus*. The numbers of parsimony-informative sites and segregating sites were also larger in *P*. *maniculatus* for all markers, except for the D-Loop, which exhibited a reverse pattern. This was also true for nucleotide diversity and mean pairwise differences among sites (Table 3).

**Table 3:** Numbers of sequences, sequence lengths (pb), numbers of haplotypes, private haplotypes, informative sites, segregating sites, nucleotide diversity and haplotype diversites for each mtDNA region used in this study for the species (sp) *P*. *leucopus* (*P*. *l*) and *P*. *maniculatus* (*P*. *m*).

Region	Sp.	Nb. Seq	Length (pb)	Nb. haplo	Private haplo	Nb. Inf. sites	Seg. sites	Nucl. div.	Haplo. div.
D-Loop	*P*. *m*	43	842	36	28	34	49	0.010	0.98
	*P*. *l*	87	839	21	11	42	51	0.017	0.89
Cyt B	*P*. *m*	43	990	27	18	32	48	0.011	0.96
	*P*. *l*	87	990	12	3	13	23	0.006	0.83
ATP8	*P*. *m*	43	158	7	3	7	9	0.016	0.72
	*P*. *l*	87	158	7	2	5	7	0.007	0.57
16S	*P*. *m*	43	294	10	5	3	8	0.004	0.59
	*P*. *l*	87	280	5	1	3	4	0.004	0.41
COIII	*P*. *m*	43	674	26	16	29	41	0.012	0.94
	*P*. *l*	87	626	11	4	15	20	0.008	0.83

**Fig 2:**
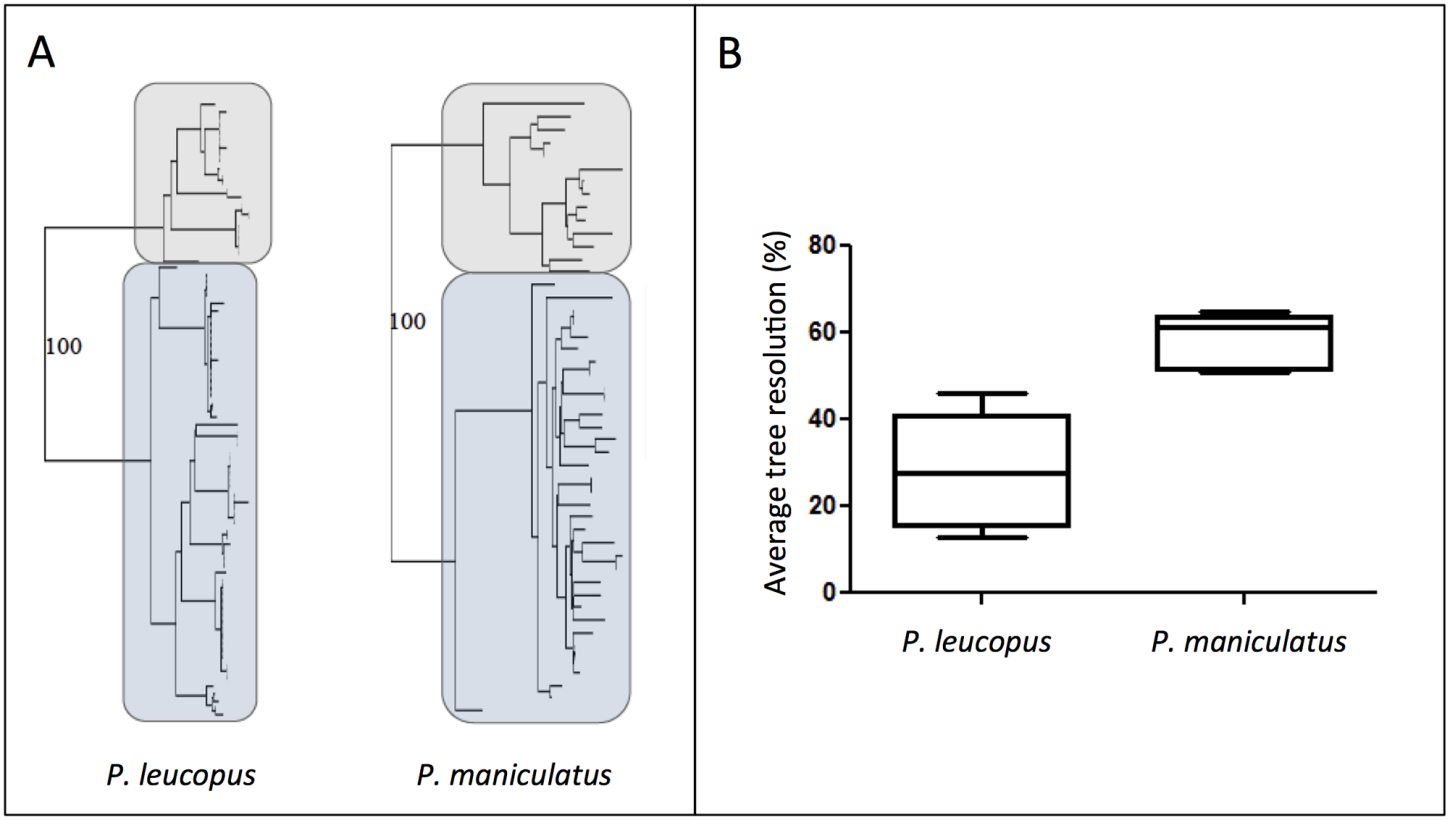
(A) Phylogeographic trees estimated by neighbor-joining from the combined sequences of 5 mtDNA regions; (B) and average topological resolution of the trees obtained for each five marker. The north-shore clades are framed in grey and the south-shore clades in blue. Boostrap support values are shown for the NS/SS split.

Results of NJ trees and PhyML trees were almost identical for all five mtDNA genes, but for some minor topological differences among terminal nodes. For both species, two major groups were well supported (BS > 95%) in all trees, and the split between the north shore (NS) and south shore (SS) of the St. Lawrence River was highly supported (S1 Fig). The population structure was a little more resolved when considering the D-Loop, Cyt B, and COIII sequences, than the 16S and the ATP8 sequences, likely due to the very small length of the later. Overall, the resolution was improved when combining all markers into a single analysis. The majority consensus of all five phylogeographic trees for each species also only contained two groups (Fig 2A), corresponding to the north and south shores. Moreover, haplotypes were more closely related in the white-footed mouse, with phylogeographic trees exhibiting a better resolution in *P*. *maniculatus* than in *P*. *leucopus* (t = 4.53, *p* < 0.01, Fig 2B). Results of incongruence length difference tests (ILD) were significant for both species (*P*. *maniculatus*: ILD = 184, *p* < 0.05; *P*. *leucopus*: ILD = 134, *p* < 0.05) Similarly, the test of congruence among distance matrix (CADM) showed that all markers were globally congruent (*P*. *maniculatus*: W = 0.633, *p* < 0.001; *P*. *leucopus*: W = 0.656, *p* < 0.001). Results of pairwise Mantel tests indicated that all markers were significantly correlated for both *Peromyscus* species (all *p* < 0.001), except for the markers ATP8 and 16S in *P*. *maniculatus* (*p* = 0.117). Based on these results, all markers were concatenated for further analysis.

**Fig 3:**
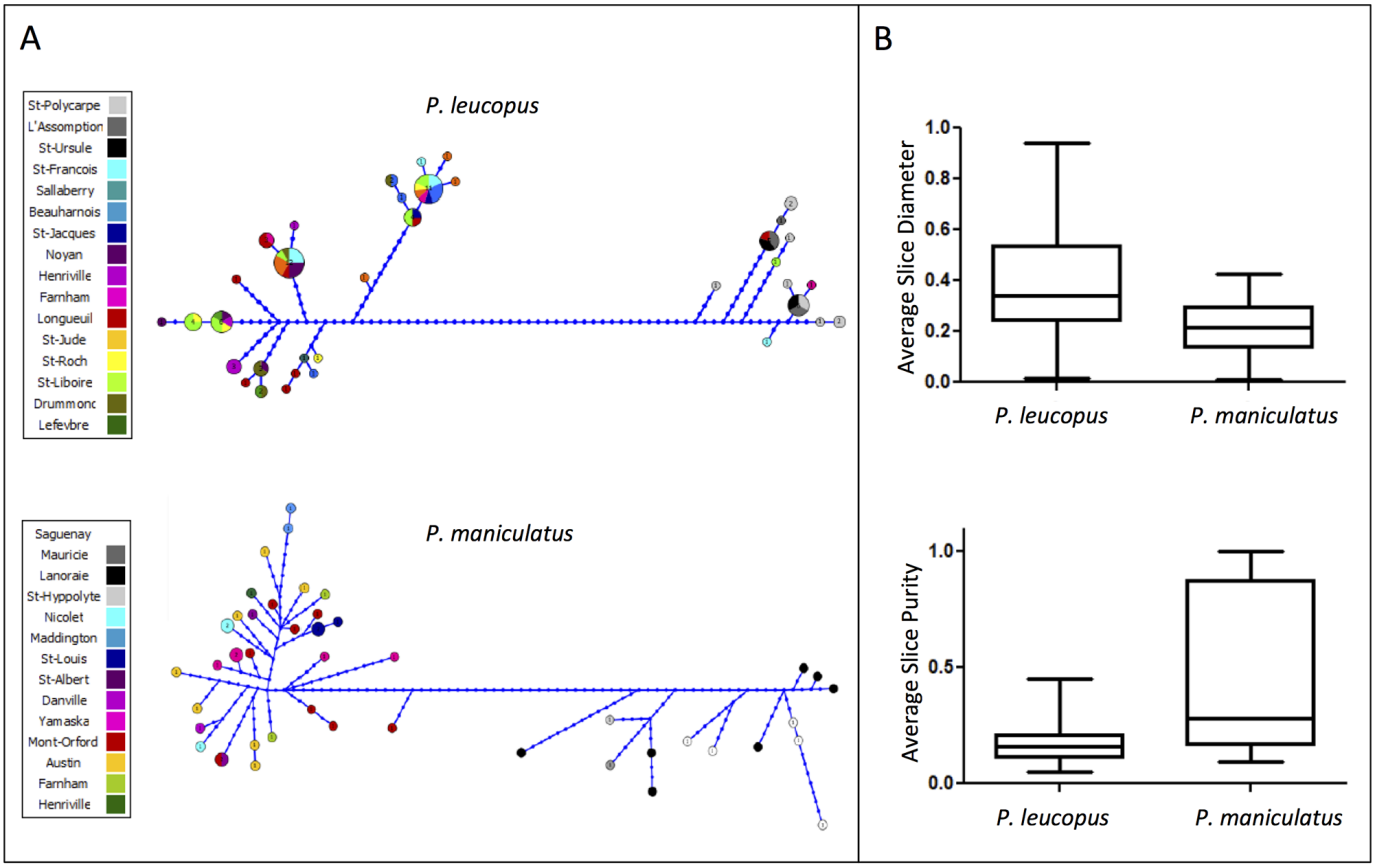
(A) Haplotype networks estimated using the combined sequences of 5 mtDNA regions for *P*. *leucopus* and *P*. *maniculatus*; (B) Associated statistics. The colors in the networks correspond to different localities where *P*. *maniculatus* and *P*. *leucopus* were sampled. For both *P*. *leucopus* and *P*. *maniculatus*, we displayed the average group (slice) diameter and group purity in the haplotype networks of sampling sites for the five markers.

**Fig 4:**
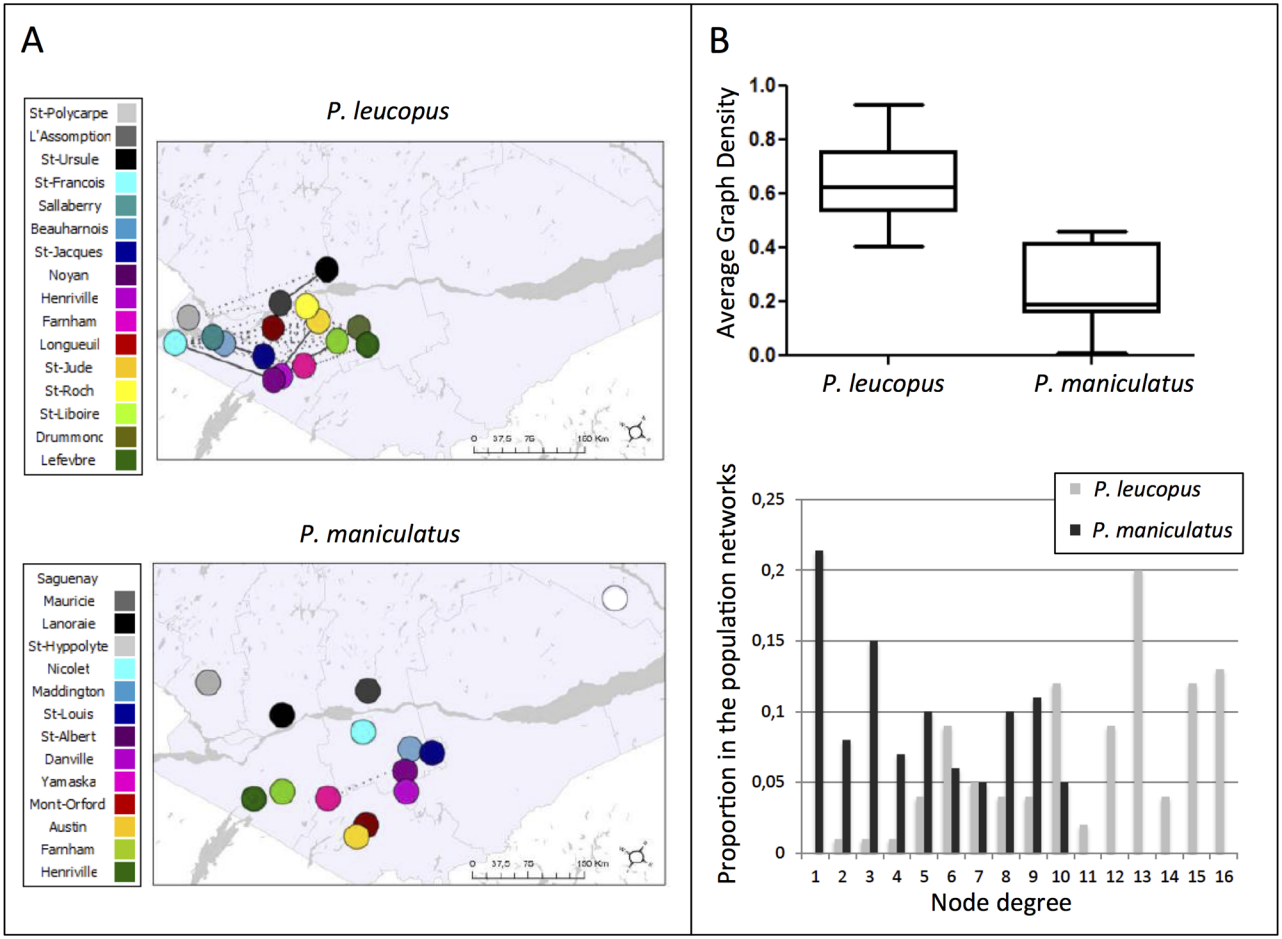
(A) Population networks using the combined sequences of 5 mtDNA regions for *P*. *leucopus* and *P*. *maniculatus*; (B) Associated statistics. The colors in the networks correspond to different localities where *P*. *maniculatus* and *P*. *leucopus* were sampled. Dotted lines correspond to connection probabilities lower than 0.2 while solid lines represent connection probabilities of 0.2 and higher. For both *P*. *leucopus* and *P*. *maniculatus*, we displayed the average graph density and the average node degree distribution for the five markers in the population networks.

The structure observed in haplotype networks indicated a larger number of shared haplotypes in *P*. *leucopus* than in *P*. *maniculatus*, for all markers combined (Fig 3A). *P*. *maniculatus* also appeared much more divergent, for each marker individually (S2 Fig). The clanistic analysis on haplotype networks showed that group diameters corresponding to each site were on average significantly larger in *P*. *leucopus* than in *P*. *maniculatus* (t = 2.51, *p* < 0.021, Fig 3B). Likewise, groups including haplotypes for each site were the purest in *P*. *maniculatus*, showing that haplotypes were shared among a larger number of sites in *P*. *leucopus*. This result was significant for slices (t = 2.53, *p* < 0.032, Fig 3B), but not for clans (t = 2.15, *p* = 0.060). Overall, connection probabilities between shared haplotypes were larger in *P*. *leucopus* than in *P*. *maniculatus* (Fig 4A). In addition, *P*. *leucopus* displayed a higher graph density for all markers combined in the population network than *P*. *maniculatus* (t = 11.39, *p* < 0.0001, Fig 4B), as well as for each marker individually (S3 Fig). *P*. *leucopus* exhibited more nodes with a high degree (larger than 3), whereas *P*. *maniculatus* displayed nodes with very few or no connections (tau = 0.557, *p* < 0.001, Fig 4B).

Values of neutrality tests were non-significant for independant mtDNA markers and all markers combined in *P*. *leucopus*, whereas several tests were significant in *P*. *maniculatus* (Table 4). Namely, all markers except ATP8 exhibited a significant departure from neutrality in deer mice sampled from the south shore of the St. Lawrence River, but a single test was significant for the north shore. Values of Tajima’s D were negative and significant for a single marker (Cyt B), while values of Ramos-Onsins and Rozas’ R_2_ and Fu’s F_s_ were respectively significant for four markers (D-Loop, Cyt B, 16S, COIII) and three markers (D-Loop, Cyt B, 16S, COIII). All three neutrality tests were significant for the south shore in *P*. *maniculatus* when all markers are combined.

**Table 4:** Results of neutrality tests for both species (*P*. *m* and *P*. *l*) and all mtDNA regions, for both shores combined or analyzed separately, with corresponding probability values (in parentheses). Significant values are in bold.

Region	Sp.	Shores	Tajima’s D	Ramos-Onsins & Rozas R_2_	Fu’s Fs
	*P*. *m*	Both	-0.84 (0.40)	0.08 (0.19)	-1.31 (0.09)
		North	-0.07 (0.94)	0.13 (0.58)	-0.09 (0.44)
D-Loop		South	-1.63 (0.10)	**0.06 (0.01)**	**-1.78 (0.05)**
	*P*. *l*	Both	1.60 (0.11)	0.15 (0.97)	1.71 (0.95)
		North	0.16 (0.88)	0.14 (0.73)	-0.56 (0.27)
		South	0.02 (0.99)	0.10 (0.63)	-0.03 (0.51)
	*P*. *m*	Both	0.02 (0.99)	0.11 (0.56)	-0.75 (0.21)
		North	-1.53 (0.13)	0.10 (0.13)	**-1.81 (0.04)**
Cyt B		South	**-2.04 (0.04)**	**0.06 (0.02)**	**-2.85 (0.01)**
	*P*. *l*	Both	1.18 (0.24)	0.14 (0.94)	1.87 (0.98)
		North	-0.32 (0.75)	0.13 (0.55)	-0.51 (0.31)
		South	-0.64 (0.52)	0.08 (0.33)	0.43 (0.70)
	*P*. *m*	Both	0.79 (0.43)	0.14 (0.85)	-0.17 (0.43)
		North	-0.96 (0.34)	0.14 (0.41)	-0.59 (0.16)
ATP8		South	-0.40 (0.69)	0.11 (0.48)	-1.30 (0.17)
	*P*. *l*	Both	-0.55 (0.58)	0.08 (0.31)	-0.60 (0.28)
		North	1.33 (0.19)	0.25 (0.96)	0.87 (0.83)
		South	-0.62 (0.53)	0.08 (0.29)	-1.17 (0.14)
	*P*. *m*	Both	-1.11 (0.27)	0.07 (0.10)	**-2.58 (0.04)**
		North	-0.20 (0.84)	0.17 (0.62)	-0.40 (0.36)
16S		South	-1.47 (0.14)	**0.07 (0.04)**	**-1.99 (0.04)**
	*P*. *l*	Both	1.09 (0.27)	0.15 (0.93)	-0.19 (0.57)
		North	1.10 (0.29)	0.23 (0.92)	0.81 (0.76)
		South	-0.44 (0.66)	0.08 (0.37)	-0.29 (0.37)
	*P*. *m*	Both	-0.38 (0.71)	0.09 (0.45)	-0.67 (0.24)
		North	-1.21 (0.23)	0.14 (0.69)	-1.51 (0.08)
COIII		South	-1.57 (0.12)	**0.06 (0.02)**	-0.94 (0.17)
	*P*. *l*	Both	0.78 (0.44)	0.12 (0.84)	0.04 (0.53)
		North	0.12 (0.90)	0.17 (0.81)	-0.52 (0.34)
		South	-0.87 (0.38)	0.07 (0.21)	-0.41 (0.35)
	*P*. *m*	Both	-0.27 (0.46)	0.10 (0.43)	-0.89 (0.17)
		North	-0.87 (0.38)	0.11 (0.17)	-1.04 (0.16)
All		South	**-1.82 (0.02)**	**0.05 (0.01)**	**-2.29 (0.03)**
	*P*. *l*	Both	1.22 (0.22)	0.14 (0.94)	1.32 (0.95)
		North	0.19 (0.85)	0.14 (0.78)	-0.46 (0.30)
		South	-0.42 (0.67)	0.09 (0.43)	-0.16 (0.45)

Analysis of mismatch distributions revealed bimodal or multimodal distributions for both species when all markers were combined (S4 Fig), suggesting that populations have been stationary for a long time. Separating the analysis for distinct shores of the St. Lawrence River produced similar multimodal distributions, except for populations of *P*. *maniculatus* located on the south shore, which exhibited a distribution with a single peak indicative of a demographic expansion.

Mean pairwise Fst values were much larger in *P*. *maniculatus* (0.619) than in *P*. *leucopus* (0.373). Out of 120 comparisons among 16 populations, 47 tests (39%) were significant for *P*. *leucoupus*, with all tests between populations located on separate shores being significant (S3 Table). Out of 78 comparisons among 13 populations, 32 tests (41%) were significant for *P*. *maniculatus*, mostly for comparisons between populations located on different shores of the St. Lawrence River (S4 Table). By restricting the comparisons only between populations from the south shore, twice as many tests were significant for *P*. *maniculatus* (24%) compared with *P*. *leucopus* (11%). Combining all populations into two groups representing the south and north shores provided significant results both for *P*. *maniculatus* (Fst = 0.776, *p* < 0.001) and P. *leucoupus* (Fst = 0.647, *p* < 0.001).

Bayesian phylogenetic analysis recovered the clades in Dragoo *et al*. [20] when our 43 Cyt B sequences for *P*. *maniculatus* were combined with the original sequences. All individuals sampled from the south shore of the St. Lawrence River fell into the east coast clade, while individuals sampled from the north shore were included in the clades from the Midwest and Eastern Canada in Dragoo *et al*. [20]. We obtained a similar pattern when the sequences published in Shipp-Penock *et al*. [19] were combined to our 87 *P*. *leucopus* D-Loop sequences. Individuals sampled from the north shore of the St. Lawrence were included in Midwestern clade, whereas those sampled from the south shore fell within the east coast clade in Shipp-Penock *et al*. [19]. Based on these results, the timing of the split between clades corresponding to the north and south shores of the St. Lawrence River is estimated at either 2.7 Mya (S5 Fig) or 2.1 Mya (S6 Fig), depending on the mtDNA marker.

## Discussion

### Congruent phylogeographic patterns

It has been shown that mtDNA markers may be biased, for instance through differential male/female dispersion rate. The use of several mtDNA markers improves the detection of significant phylogeographic signals, and they can display patterns congruent with those found using nuclear markers [65, 66]. Here, we found that the phylogeographic structure was congruent among the five mtDNA markers we used. For both *Peromyscus* species, samples from the south shore of the St. Lawrence River and samples from the north shore fell in distinct groups, and results of Fst tests between both shores were significant, which confirms our hypothesis that the St. Lawrence River constitutes a major geographical barrier to dispersal for *Peromyscus* individuals. Besides this strong separation, we found no further clear spatial structure in our data. Individuals from a given site were seldom grouped together, especially in *P*. *leucopus*.

Different mtDNA sequences may also exhibit varying degrees of ancestral polymorphism and thus not reflect consistent coalescent histories [66], which supports the need for considering more than a single marker in phylogeographic studies. Here, we found that the variability of the COIII marker was similar to that found in another phylogeographic study on *P*. *leucopus* and *P*. *maniculatus* in Québec [67]. The variability level observed in *P*. *eremicus* in Baja California for the Cyt B and COIII was about ten times larger than for *P*. *leucopus* and *P*. *maniculatus* in Québec, likely because the former study included samples from a much larger geographical area [22]. Likewise, Shipp-Pennock *et al*. [19] obtained sequences twice as variable as ours for *P*. *leucopus* using the D-Loop in a study that included specimens sampled throughout the eastern United States and Canada. With respect to the numbers of haplotypes, other studies have identified approximately ten times more than we did using the D-Loop in *P*. *leucopus* across the United-Sates [18]. However, we found proportionally more haplotypes than Dragoo *et al*. [20], based on Cyt B sequences of *P*. *maniculatus*. These results illustrate the benefits of using multiple markers at a smaller geographical scale, but also show that different mtDNA regions may behave differently in different species.

### Range expansion and demographic changes

We detected the signature of a recent expansion of white-footed mouse populations that displayed a significantly distinct genetic pattern than the one observed in the deer mouse, which has long been established in Québec. Haplotype network analyses clearly showed that *P*. *maniculatus* was more structured across the sampling area than *P*. *leucopus*. Namely, haplotypes from the same site tended to be more clustered with respect to localities in *P*. *maniculatus* than in *P*. *leucopus*. Most haplotypes defined by the combination of all markers were unique in *P*. *maniculatus*, while *P*. *leucopus* exhibited a large number of shared haplotypes between sites. Shared haplotypes are generally considered as ancestral retention and provide evidence for continuous migration between populations [10, 68].

Using population network analyses we also found that *P*. *leucopus* had widespread haplotypes without any barrier other than the St. Lawrence River, whereas *P*. *maniculatus* only shared a few haplotypes between sites. These results are consistent with a recent northern expansion of white-footed mice detected in Michigan [9] and in Southern Québec [16]. Similarly, by comparing the phylogeographic patterns of twelve sympatric species of frogs, the genetic homogeneity observed throughout the sampling area was attributed to a recent range expansion during favorable climatic conditions [66].

The low genetic diversity we observed in *P*. *leucopus* in Southern Québec is consistent with a recent colonisation and a rapid range expansion [69]. On the other hand, the high diversity of closely related haplotypes with no clear geographic structure and the lack of apparent gene flow in *P*. *maniculatus* confirm that these populations have established in the region for a longer time, an observation corroborated by other field studies [9]. Populations of *P*. *maniculatus* appeared to be more structured and more diversified than populations of *P*. *leucopus* sampled in the study area. This clear phylogeographic structure suggests that *P*. *maniculatus* reproduces mostly within established populations, and that gene flow between these populations is reduced compared with *P*. *leucoupus*. Interestingly, neutrality tests revealed a significant departure from neutrality in populations of *P*. *maniculatus* sampled from the south shore of the St. Lawrence River. The same populations of *P*. *maniculatus* also exhibited a mismatch distribution with a unique mode. This was not observed on the north shore, and not for *P*. *leucopus*, however. Significant deviations from neutrality can be explained by selection or demographic fluctuations such as population expansion following a bottleneck [59]. Likewise, populations that have been experiencing a recent demographic expansion [57, 70] or range expansion [71, 72] generate unimodal mismatch distributions. In the present case, we postulate that interspecific competition between the two *Peromyscus* species [73, 74] could result in a selective sweep of deer mouse populations on the south shore of the St. Lawrence River, following the recent northward range expansion of the white-footed mouse [9, 16]. These results further reinforce the difference in their phylogeographic structures, especially if *P*. *maniculatus* were extirpated from territory newly colonized by *P*. *leucopus*.

Overall, our results are consistent with the hypothesis of a range expansion in *P*. *leucopus*, and have important implications for the spread of Lyme disease in southern Québec, as the risk of occurrence of *B*. *burgdorferi* was strongly associated with the presence of *P*. *leucopus* in our study area [27, 29].

### Distinct lineages and implications for the emergence of Lyme disease

All five markers used in this study supported an ancestral divergence of mouse populations from the north shore of the St. Lawrence River and populations from the south shore, in both *P*. *leucopus* and *P*. *maniculatus*. When the sequences of the two species sampled in southern Québec were combined with those obtained in previous studies across North America [19, 20], we found that mice from both shores of the St. Lawrence River belonged to different ancestral clades, in the two species. Mice from the north shore of the St. Lawrence River originated from a Midwestern clade, while mice from the South shore originated from the East Coast clade. Previous studies have also shown that these clades represent different glacial refugia that have since then been expanding northwards independently [18]. Based on our results, the split between these clades corresponding to distinct shores of the St. Lawrence River occurred well before the Late Glacial Maximum. We estimated the time of this divergence at around 2.1–2.7 Mya, a value that must be taken with caution since we did not consider a third *Peromyscus* species as an outgroup for this analysis.

The St. Lawrence River thus appears to be a geographic barrier sufficiently strong to maintain the ancestral divergence between the two distinct lineages of *P*. *leucopus* in Québec, and likely distinct associated strains of *B*. *burgdorferi*. In line with this hypothesis, a phylogeographic pattern similar to that observed for the mice was detected in *B*. *burgdorferi*, with two main lineages of *Borrelia* (Midwest and North-East) that diverged since the Late Glacial Maximum across North America [75, 76]. These lineages possess different forms of the OspC protein, an antigen against which mammals can develop immunity [77]. Experimental studies have shown that *P*. *leucopus* immunity to *B*. *burgdorferi* is strain specific [78]. Since distinct lineages of *B*. *burgdorferi* geographically match distinct clades of *P*. *leucopus* in the United States, the two different clades of *P*. *leucopus* found in Québec are likely carrying distinct strains of the bacteria. We expect two strains of *B*. *burgdorferi*, that diverged since the Late Glacial Maximum, to prevail in Québec and that these strains will keep on spreading with the steady range expansion of *P*. *leucopus* in the province. It has been shown that *B*. *burgdorferi* subspecies manifest different clinical symptoms and responses to diagnosis in humans [79]. The existence of different virulence levels associated with different strains of *B*. *burgdorferi* and distinct clades of *P*. *leucopus* has significant implications for the monitoring of the emergence of Lyme disease in Québec, and the design of efficient strategies to lower the impact of this zoonosis on human populations.

## Supporting Information

S1 Fig Phylogeographic trees constructed by neighbor-joining from 5 mitochondrial genes (D-loop, Cyt B, ATP8, 16S and COIII) for *P*. *leucopus* and *P*. *maniculatus*. The north-shore (NS) clade is framed in grey and the south-shore (SS) in blue. Boostrap support values are shown for the NS/SS split.
(TIF)

S2 Fig Haplotype network estimated from 5 mitochondrial genes, for *P*. *leucopus* and *P*. *maniculatus*. The colors in the networks correspond to sampling localities. North shore sites are on a grey-scale while south shore sites are in color.
(TIF)

S3 Fig Population network estimated from 5 mitochondrial genes, for *P*. *leucopus* and *P*. *maniculatus*. The colors in the networks correspond to sampling localities. North shore sites are on a grey-scale while south shore sites are in color. Dotted lines correspond to connection probabilities lower than 0.2 while solid lines represent connection probabilities of 0.2 and higher.
(TIF)

S4 Fig Mismatch distribution analysis for *P*. *leucopus* and *P*. *maniculatus* based on all markers combined. Distributions are presented for populations located (a) on both shores, (b) only the north shore, and (c) only the south shore of the St. Lawrence River. Empirical distributions (in blue) and expected distributions under a stable population model (in red) are shown on each plot.
(TIF)

S5 Fig Bayesian phylogenetic tree based on the 87 D-Loop sequences of *Peromyscus leucopus* included in the present study combined with the sequences in Shipp-Pennock *et al* [20]. To calibrate the molecular clock, the corresponding 43 sequences of *Peromyscus maniculatus* were used as outgroup, and the split between both species was set at 3.5 Mya. Clades corresponding to the north and south shores of St. Lawrence River are highlighted to estimate their divergence time.
(TIF)

S6 Fig Bayesian phylogenetic tree based on the 43 Cyt B sequences of *Peromyscus maniculatus* included in the present study combined with the sequences in Dragoo *et al*. [20]. To calibrate the molecular clock, the corresponding 87 sequences of *Peromyscus leucopus* were used as outgroup, and the split between both species was set at 3.5 Mya. Clades corresponding to the north and south shores of St. Lawrence River are highlighted to estimate their divergence time.
(TIF)

S1 Table Alignments of 87 *Peromyscus leucopus* sequences for all mtDNA regions combined. (TXT)

S2 Table Alignments of 43 *Peromyscus maniculatus* sequences for all mtDNA regions combined. (TXT)

S3 Table Pairwise Fst values (above diagonal) and corresponding probabilities (below diagonal) among the 16 populations of *Peromyscus leucopus* sampled in this study. Significant Fst values are in bold.
(DOCX)

S4 Table Pairwise Fst values (above diagonal) and corresponding probabilities (below diagonal) among the 13 populations of *Peromyscus maniculatus* sampled in this study. Significant Fst values are in bold.
(DOCX)
