# Supplementary figures and images for: Phylogeographic Structure of the White-Footed Mouse and the Deer Mouse, Two Lyme Disease Reservoir Hosts in Québec
Source: PLoS One. 2015 Dec 3;10(12):e0144112. doi: 10.1371/journal.pone.0144112 (PMC4669108; doi:10.1371/journal.pone.0144112)

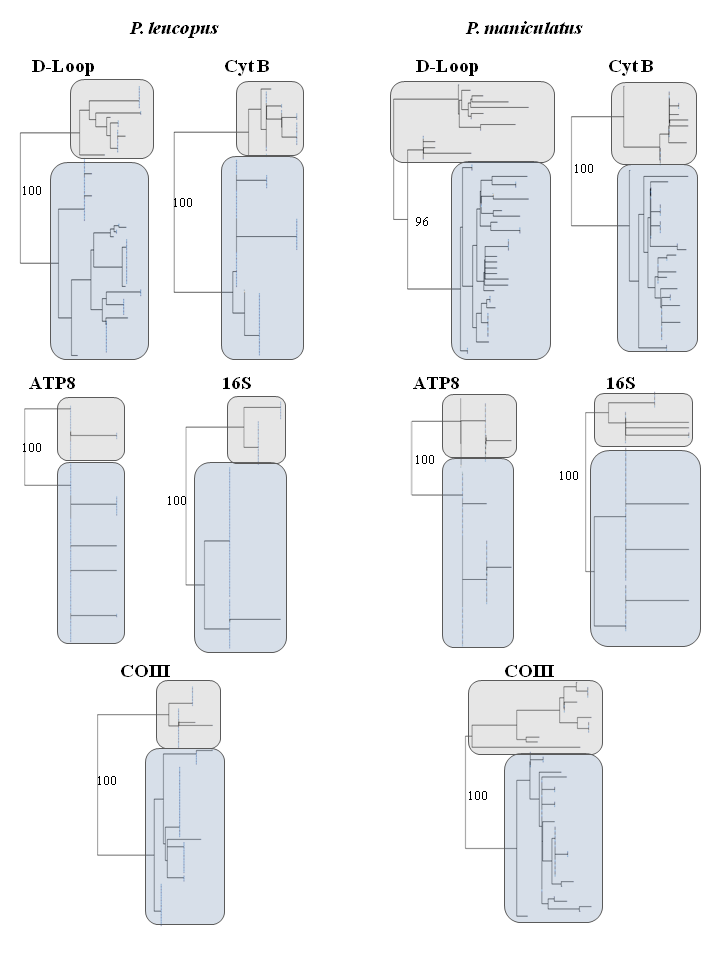

Supplement: S1 Fig — The north-shore (NS) clade is framed in grey and the south-shore (SS) in blue. Boostrap support values are shown for the NS/SS split. (TIF) [file pone.0144112.s001.tif]

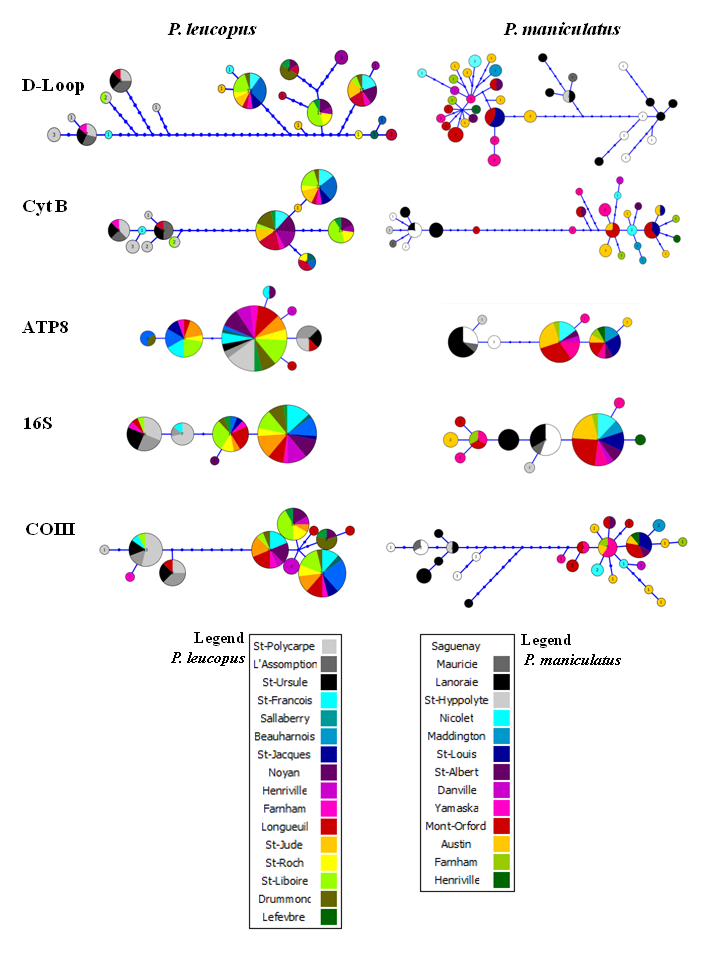

Supplement: S2 Fig — The colors in the networks correspond to sampling localities. North shore sites are on a grey-scale while south shore sites are in color. (TIF) [file pone.0144112.s002.tif]

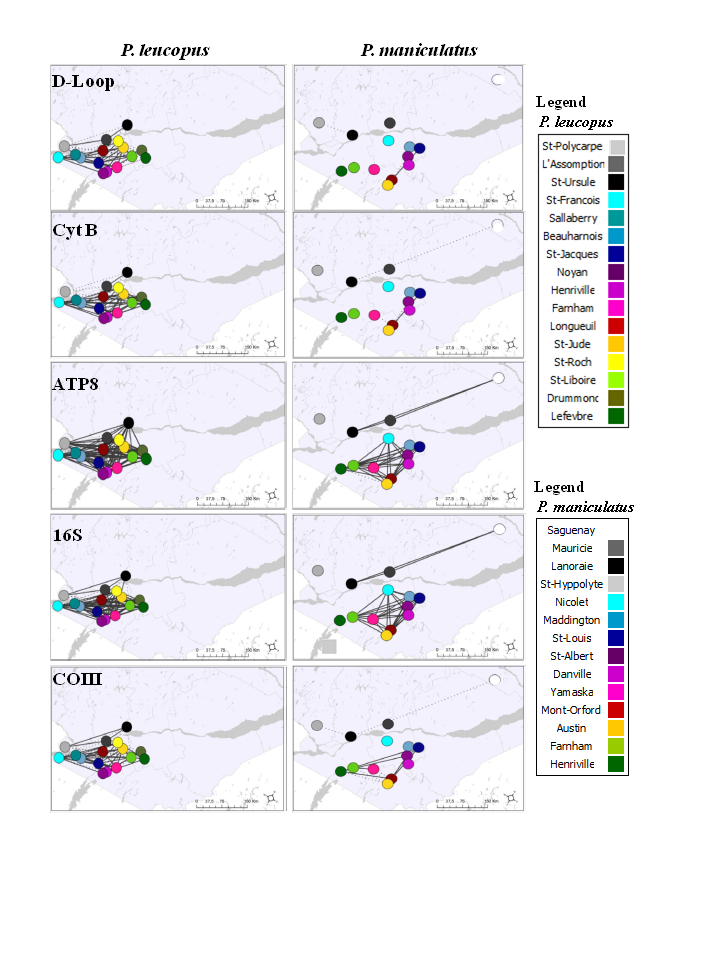

Supplement: S3 Fig — The colors in the networks correspond to sampling localities. North shore sites are on a grey-scale while south shore sites are in color. Dotted lines correspond to connection probabilities lower than 0.2 while solid lines represent connection probabilities of 0.2 and higher. (TIF) [file pone.0144112.s003.tif]

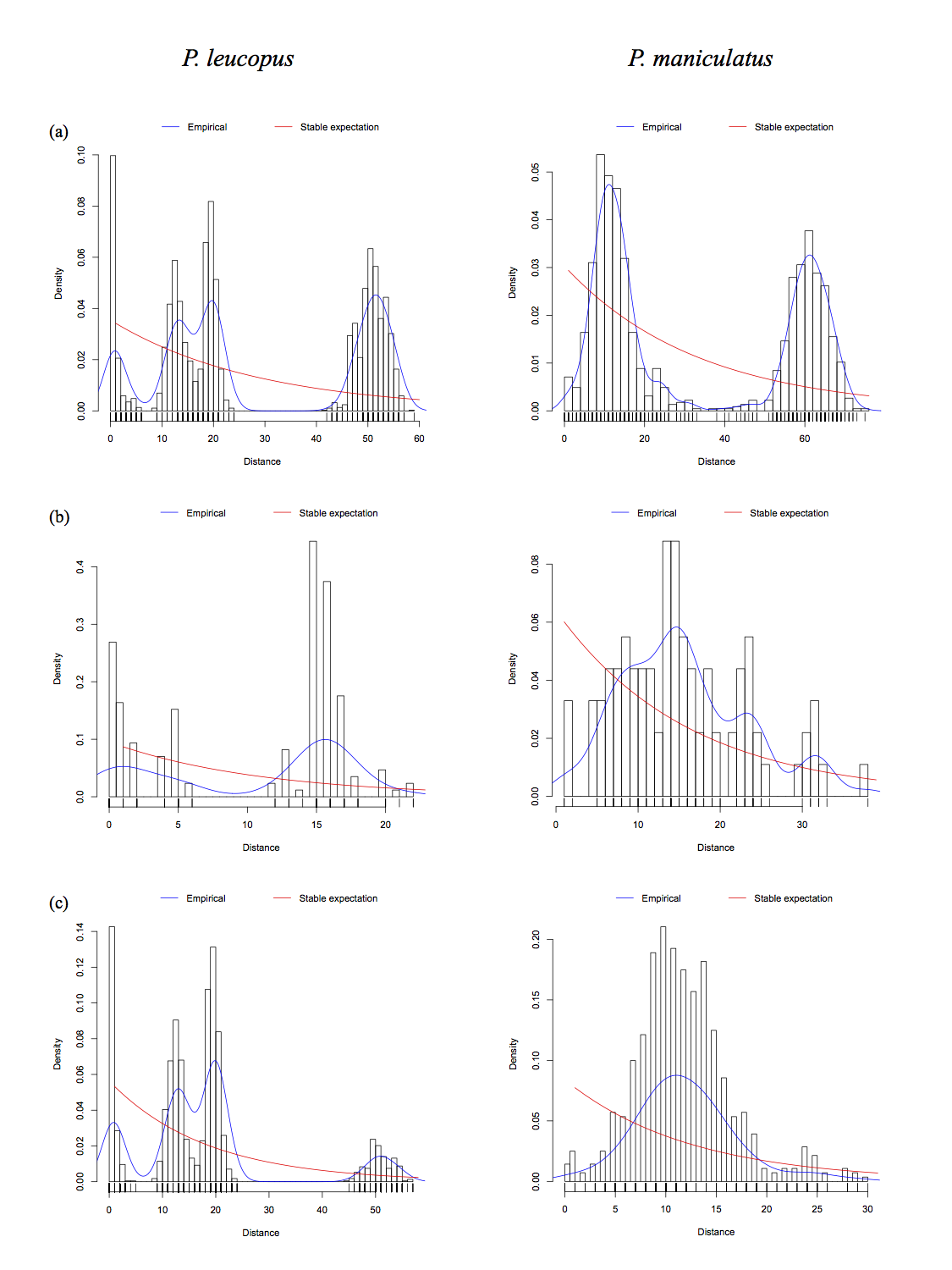

Supplement: S4 Fig — Distributions are presented for populations located (a) on both shores, (b) only the north shore, and (c) only the south shore of the St. Lawrence River. Empirical distributions (in blue) and expected distributions under a stable population model (in red) are shown on each plot. (TIF) [file pone.0144112.s004.tif]

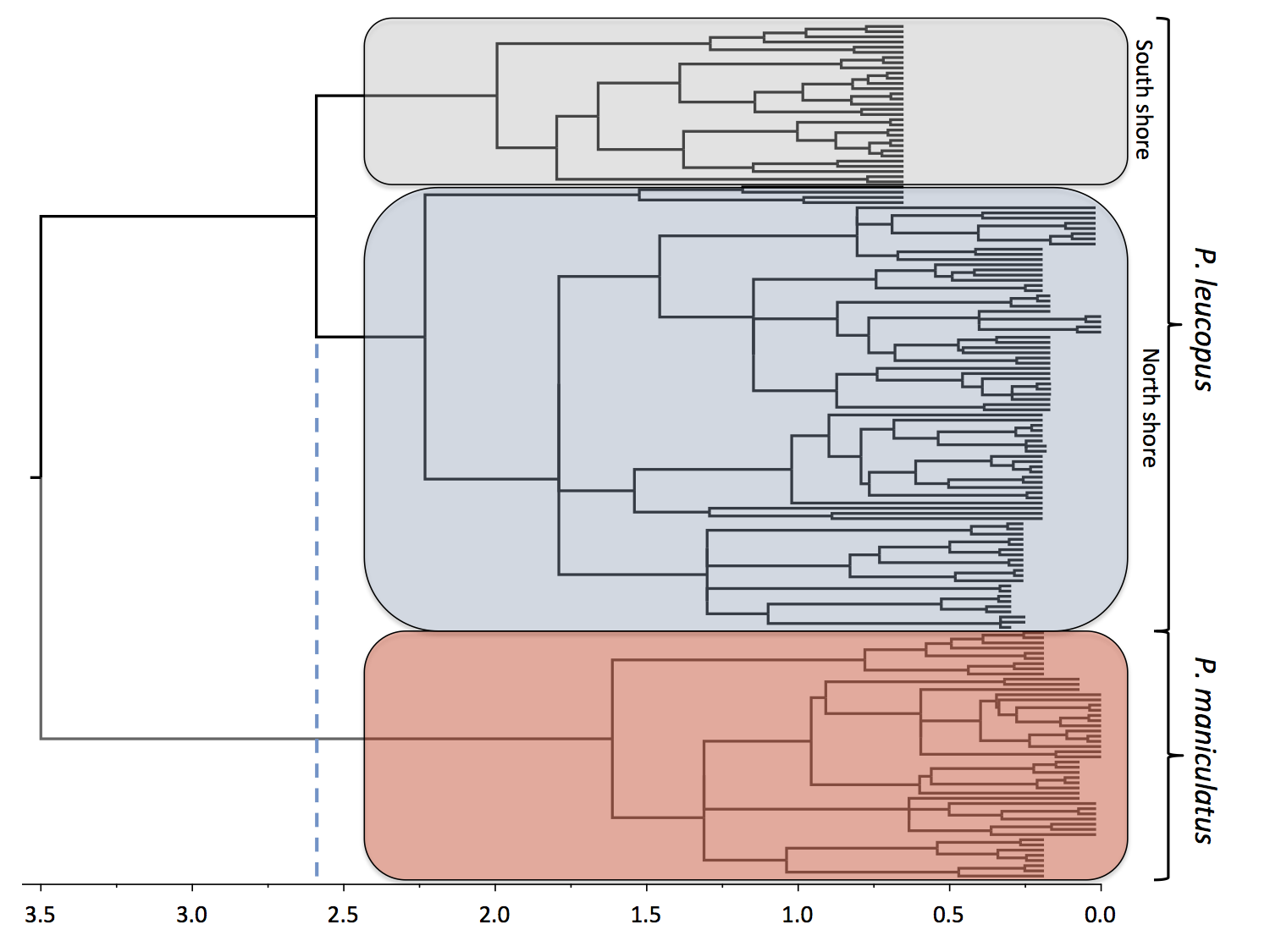

Supplement: S5 Fig — To calibrate the molecular clock, the corresponding 43 sequences of Peromyscus maniculatus were used as outgroup, and the split between both species was set at 3.5 Mya. Clades corresponding to the north and south shores of St. Lawrence River are highlighted to estimate their divergence time. (TIF) [file pone.0144112.s005.tif]

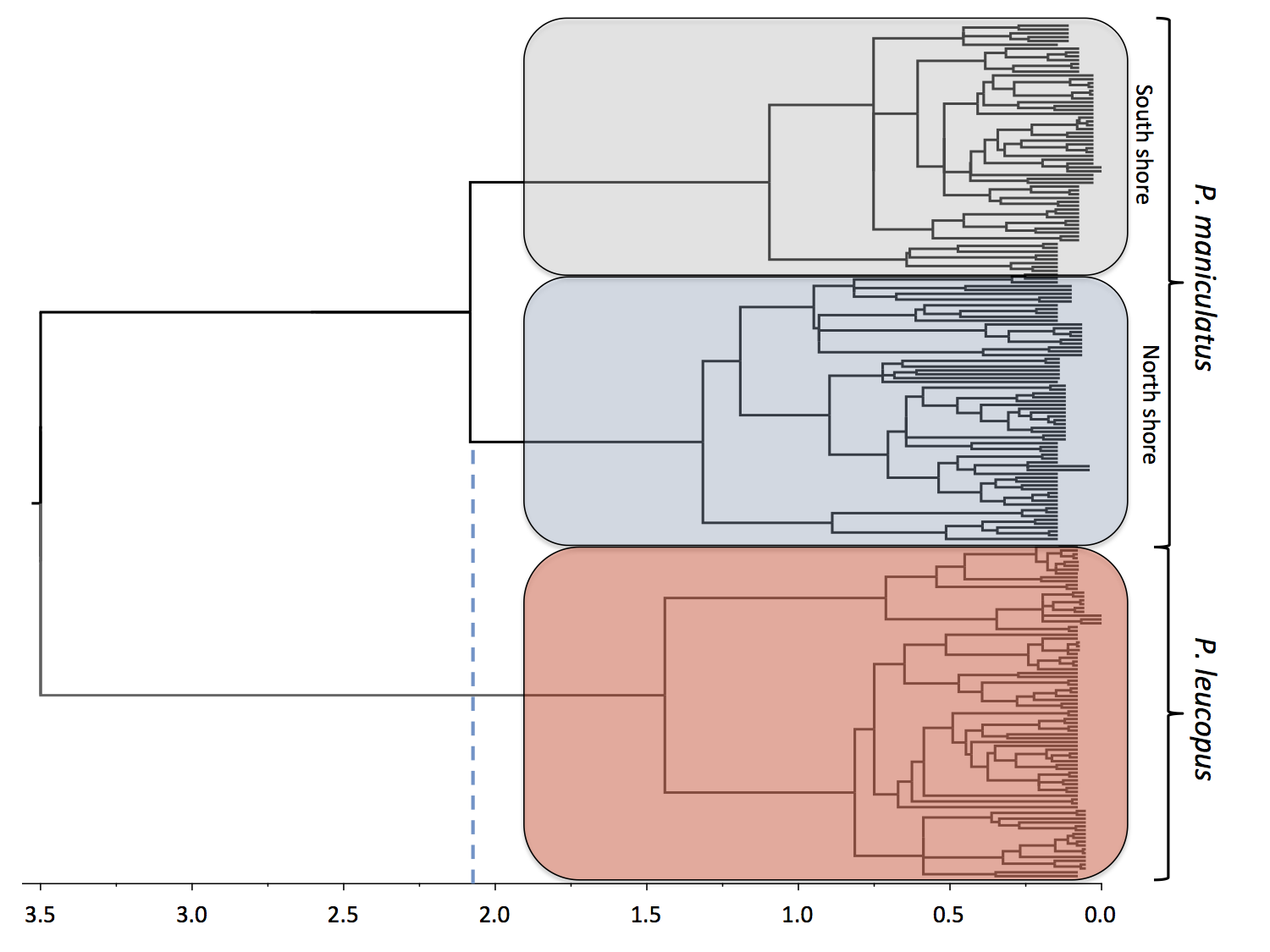

Supplement: S6 Fig — To calibrate the molecular clock, the corresponding 87 sequences of Peromyscus leucopus were used as outgroup, and the split between both species was set at 3.5 Mya. Clades corresponding to the north and south shores of St. Lawrence River are highlighted to estimate their divergence time. (TIF) [file pone.0144112.s006.tif]
