# Supplementary material for: Phylogeographic Structure of the White-Footed Mouse and the Deer Mouse, Two Lyme Disease Reservoir Hosts in Québec
Source: PLoS One. 2015 Dec 3;10(12):e0144112. doi: 10.1371/journal.pone.0144112 (PMC4669108; doi:10.1371/journal.pone.0144112)
Supplement: S3 Table — Significant Fst values are in bold. (DOCX) [file pone.0144112.s009.docx]

|  |  | 1 | | 2 | | 3 | | 4 | | 5 | | 6 | | 7 | | 8 | | 9 | | 10 | | 11 | | 12 | | 13 | | 14 | | 15 | | 16 | |  |
| --- | --- | --- | --- | --- | --- | --- | --- | --- | --- | --- | --- | --- | --- | --- | --- | --- | --- | --- | --- | --- | --- | --- | --- | --- | --- | --- | --- | --- | --- | --- | --- | --- | --- | --- |
| 1 | St-Polycarpe | |  | | 0.06 | | -0.02 | | **0.62** | | **0.89** | | **0.81** | | **0.87** | | **0.80** | | **0.80** | | **0.29** | | **0.65** | | **0.77** | | **0.75** | | **0.67** | | **0.77** | | **0.77** | |
| 2 | L'Assomption | | 0.25 | |  | | -0.26 | | **0.65** | | **0.91** | | **0.84** | | **0.90** | | **0.81** | | **0.81** | | **0.34** | | **0.66** | | **0.79** | | **0.77** | | **0.70** | | **0.79** | | **0.79** | |
| 3 | St-Ursule | | 0.42 | | 1.00 | |  | | **0.64** | | **0.90** | | **0.83** | | **0.89** | | **0.81** | | **0.81** | | **0.31** | | **0.66** | | **0.78** | | **0.76** | | **0.69** | | **0.78** | | **0.78** | |
| 4 | St-Francois | | 0.01 | | 0.01 | | 0.01 | |  | | 0.41 | | 0.09 | | 0.23 | | 0.15 | | 0.25 | | 0.25 | | -0.02 | | -0.09 | | 0.06 | | 0.00 | | 0.09 | | 0.20 | |
| 5 | Sallaberry | | 0.01 | | 0.01 | | 0.04 | | 0.55 | |  | | 0.77 | | 0.97 | | 0.58 | | 0.65 | | 0.24 | | 0.28 | | 0.57 | | 0.39 | | 0.46 | | 0.55 | | 0.54 | |
| 6 | Beauharnois | | 0.01 | | 0.01 | | 0.01 | | 0.22 | | 0.07 | |  | | -0.03 | | **0.56** | | **0.59** | | 0.00 | | **0.31** | | 0.15 | | 0.28 | | 0.20 | | **0.40** | | 0.51 | |
| 7 | St-Jacques | | 0.01 | | 0.01 | | 0.01 | | 0.29 | | 0.18 | | 0.53 | |  | | **0.73** | | **0.75** | | 0.09 | | 0.47 | | 0.37 | | 0.48 | | 0.35 | | **0.60** | | 0.68 | |
| 8 | Noyan | | 0.01 | | 0.01 | | 0.01 | | 0.12 | | 0.24 | | 0.01 | | 0.02 | |  | | 0.13 | | 0.07 | | 0.01 | | 0.18 | | 0.02 | | 0.11 | | 0.07 | | -0.15 | |
| 9 | Henriville | | 0.01 | | 0.01 | | 0.01 | | 0.06 | | 0.18 | | 0.01 | | 0.02 | | 0.16 | |  | | 0.13 | | 0.13 | | **0.32** | | 0.13 | | 0.18 | | 0.19 | | -0.02 | |
| 10 | Farnham | | 0.04 | | 0.05 | | 0.05 | | 0.96 | | 0.79 | | 0.35 | | 0.35 | | 0.25 | | 0.18 | |  | | -0.13 | | -0.11 | | -0.02 | | -0.10 | | 0.00 | | 0.08 | |
| 11 | Longueuil | | 0.01 | | 0.01 | | 0.01 | | 0.55 | | 0.97 | | 0.03 | | 0.10 | | 0.41 | | 0.21 | | 0.58 | |  | | 0.03 | | 0.01 | | 0.04 | | 0.02 | | 0.04 | |
| 12 | St-Jude | | 0.01 | | 0.01 | | 0.01 | | 0.82 | | 0.29 | | 0.06 | | 0.16 | | 0.10 | | 0.02 | | 0.59 | | 0.28 | |  | | 0.05 | | 0.04 | | 0.12 | | 0.24 | |
| 13 | St-Roch | | 0.01 | | 0.01 | | 0.01 | | 0.35 | | 0.55 | | 0.06 | | 0.10 | | 0.45 | | 0.30 | | 0.36 | | 0.38 | | 0.28 | |  | | -0.12 | | 0.07 | | -0.18 | |
| 14 | St-Liboire | | 0.01 | | 0.01 | | 0.02 | | 0.33 | | 0.42 | | 0.06 | | 0.26 | | 0.13 | | 0.10 | | 0.55 | | 0.25 | | 0.28 | | 0.76 | |  | | 0.11 | | 0.00 | |
| 15 | Drummond | | 0.01 | | 0.01 | | 0.01 | | 0.21 | | 0.22 | | 0.01 | | 0.05 | | 0.34 | | 0.12 | | 0.36 | | 0.37 | | 0.15 | | 0.35 | | 0.16 | |  | | -0.16 | |
| 16 | Lefebvre | | 0.02 | | 0.01 | | 0.03 | | 0.21 | | 0.35 | | 0.10 | | 0.08 | | 0.55 | | 0.40 | | 0.24 | | 0.34 | | 0.18 | | 0.68 | | 0.45 | | 0.58 | |  | |
