# Supplementary material for: Phylogeographic Structure of the White-Footed Mouse and the Deer Mouse, Two Lyme Disease Reservoir Hosts in Québec
Source: PLoS One. 2015 Dec 3;10(12):e0144112. doi: 10.1371/journal.pone.0144112 (PMC4669108; doi:10.1371/journal.pone.0144112)
Supplement: S4 Table — Significant Fst values are in bold. (DOCX) [file pone.0144112.s010.docx]

|  |  | 1 | 2 | 3 | 4 | 5 | 6 | 7 | 8 | 9 | 10 | 11 | 12 | 13 |
| --- | --- | --- | --- | --- | --- | --- | --- | --- | --- | --- | --- | --- | --- | --- |
| 1 | Mauricie |  | 0.53 | 1.00 | 0.93 | **0.99** | **0.99** | 0.88 | 1.00 | **0.92** | **0.90** | **0.90** | 0.89 | 1.00 |
| 2 | Lanoraie | 0.32 |  | 0.49 | **0.81** | **0.88** | **0.88** | 0.76 | 0.88 | **0.79** | **0.77** | **0.78** | **0.77** | **0.89** |
| 3 | St-Hyppolyte | 1.00 | 0.32 |  | **0.93** | **0.99** | **0.99** | 0.87 | 1.00 | **0.91** | **0.89** | **0.90** | 0.88 | 1.00 |
| 4 | Nicolet | 0.06 | 0.01 | 0.03 |  | **0.66** | **0.64** | 0.03 | 0.55 | 0.26 | 0.21 | 0.15 | 0.08 | 0.67 |
| 5 | Maddington | 0.03 | 0.01 | 0.03 | 0.04 |  | **0.92** | 0.38 | **0.97** | **0.65** | **0.56** | **0.52** | 0.40 | **0.95** |
| 6 | St-Louis | 0.01 | 0.01 | 0.01 | 0.02 | 0.01 |  | 0.20 | **0.98** | **0.50** | 0.35 | 0.39 | 0.08 | **0.94** |
| 7 | St-Albert | 0.19 | 0.02 | 0.16 | 0.39 | 0.12 | 0.25 |  | 0.48 | -0.05 | -0.14 | -0.09 | -0.21 | 0.35 |
| 8 | Danville | 1.00 | 0.03 | 1.00 | 0.26 | 0.03 | 0.01 | 0.36 |  | 0.64 | 0.59 | 0.56 | 0.50 | 1.00 |
| 9 | Yamaska | 0.01 | 0.01 | 0.01 | 0.08 | 0.02 | 0.03 | 0.54 | 0.18 |  | 0.00 | 0.09 | -0.02 | 0.62 |
| 10 | Mont-Orford | 0.01 | 0.01 | 0.02 | 0.14 | 0.02 | 0.06 | 0.65 | 0.19 | 0.34 |  | 0.05 | -0.09 | 0.48 |
| 11 | Austin | 0.04 | 0.01 | 0.03 | 0.20 | 0.02 | 0.06 | 0.55 | 0.30 | 0.23 | 0.28 |  | -0.12 | 0.51 |
| 12 | Farnham | 0.14 | 0.03 | 0.20 | 0.24 | 0.08 | 0.28 | 0.84 | 0.38 | 0.50 | 0.67 | 0.73 |  | 0.30 |
| 13 | Henriville | 1.00 | 0.05 | 1.00 | 0.18 | 0.04 | 0.02 | 0.52 | 1.00 | 0.17 | 0.40 | 0.36 | 0.58 |  |
